# Prescription of Castor Oil

**Published:** 1846-07

**Authors:** 


*Prescription of Castor Oil.*—It is worth knowing that the nauseous greasy taste of castor oil is pretty effectually disguised by mixing it with milk, and adding a little nitric æther and oil of cinnamon.—*Druitt.*

